# Scalable super hygroscopic polymer films for sustainable moisture harvesting in arid environments

**DOI:** 10.1038/s41467-022-30505-2

**Published:** 2022-05-19

**Authors:** Youhong Guo, Weixin Guan, Chuxin Lei, Hengyi Lu, Wen Shi, Guihua Yu

**Affiliations:** Materials Science and Engineering Program and Walker Department of Mechanical Engineering, The University of Texas at Austin, Austin, TX 78712 USA

**Keywords:** Polymers, Gels and hydrogels, Devices for energy harvesting

## Abstract

Extracting ubiquitous atmospheric water is a sustainable strategy to enable decentralized access to safely managed water but remains challenging due to its limited daily water output at low relative humidity (≤30% RH). Here, we report super hygroscopic polymer films (SHPFs) composed of renewable biomasses and hygroscopic salt, exhibiting high water uptake of 0.64–0.96 g g^−1^ at 15–30% RH. Konjac glucomannan facilitates the highly porous structures with enlarged air-polymer interfaces for active moisture capture and water vapor transport. Thermoresponsive hydroxypropyl cellulose enables phase transition at a low temperature to assist the release of collected water via hydrophobic interactions. With rapid sorption-desorption kinetics, SHPFs operate 14–24 cycles per day in arid environments, equivalent to a water yield of 5.8–13.3 L kg^−1^. Synthesized via a simple casting method using sustainable raw materials, SHPFs highlight the potential for low-cost and scalable atmospheric water harvesting technology to mitigate the global water crisis.

## Abstract (web summary)

Extracting atmospheric water is a sustainable strategy to enable decentralized access to safely managed water but remains impractical due to its limited daily water output at low relative humidity. Here, the authors demonstrate a hygroscopic polymer composed of renewable biomass which allows high water uptake at low relative humidity

## Introduction

**Fig. 1:**
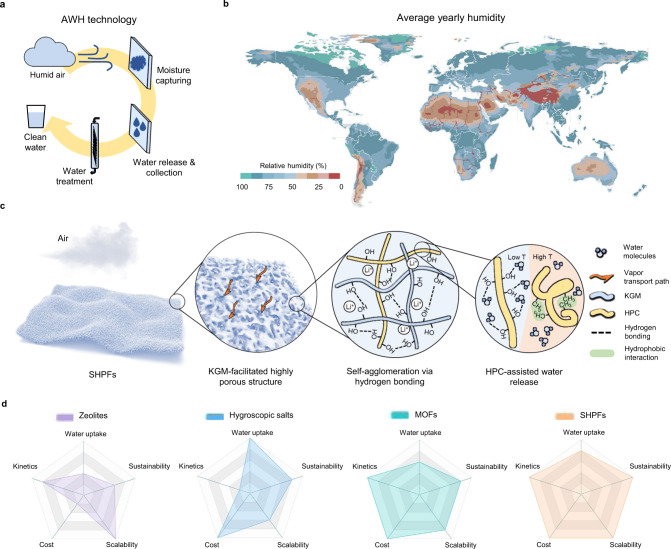
AWH technology and design principle of SHPFs. **a** Key steps of AWH technology. **b** Geographic distribution of world average annual relative humidity^9^. Regions with less than 40% RH are indicated from brown to red (warm color) regions. **c** Material design of SHPFs for AWH at low RH. **d** Qualitative comparison of different materials in terms of core requirements for practical application of AWH.

Two-thirds of the global population is experiencing water scarcity at various levels^1^. While numerous research efforts have been made to develop desalination and water purification technologies^2^, the moisture in the atmosphere, which is estimated to be more than ten thousand cubic kilometers, is another sustainable source of freshwater that can be harvested to mitigate the current water shortage^3^. Compared to conventional water purification technologies that rely on the existence of a waterbody, the extraction of water from air represents a decentralized approach regardless of geographical or hydrologic conditions^4^. The key steps of atmospheric water harvesting (AWH) involve moisture capture and water release, followed by a simple filtration or purification process (Fig. 1a). The earlier approaches, such as fog capturing^5,6^ and dew condensation^7,8^, require the presence of high relative humidity (RH) (>90% RH), which is not a viable solution considering more than one-third of the global terrestrial area has an average annual humidity less than 40% (Fig. 1b)^9^.

Porous sorbent materials have been exploited for AWH over a wide range of humidity, especially under low RH (≤30%)^10^. For instance, zeolites^11^ and silica gels^12^ with large surface areas showed vapor adsorption capabilities over a wide humidity range. However, they commonly suffer from low water uptake and high energy demand for water desorption. Other hygroscopic salts, such as LiCl, CaCl_2_, and MgCl_2_, presented higher water uptakes at low RH. However, the aggregation of salt crystals during hydration leads to the formation of passivation layers, resulting in the sluggish kinetics and decay of cycling performance^13^. As such, salt composites have been developed to improve the kinetics, immobilize salts during deliquescence, and prevent salt leakage^14–16^. Lately, metal-organic frameworks have been demonstrated with a promising moisture capture capability at low RH with relatively fast kinetics^17–19^.

Alternatively, polymeric gels have emerged as another promising platform to enable high water uptake at low RH due to their high water retention, tuneable structures, and tailorable polymer–water interactions^20–22^. In this work, we develop super hygroscopic polymer films (SHPFs) to extract water vapor from arid climates (≤30% RH) with exceptional kinetics. The SHPF consists of earth-abundant biomasses, konjac glucomannan (KGM), and hydroxypropyl cellulose (HPC) as the hybrid polymer matrix to hold uniformly dispersed LiCl solution (Fig. 1c), achieving 0.64 and 0.96 g g^–1^ water uptake at 15% and 30% RH, respectively. Specifically, SHPFs have hierarchically porous structures facilitated by KGM, which provide enlarged sorbent-air interfaces and rapid water vapor transport pathways. Thermoresponsive HPC permits controllable interactions between polymer chains and water molecules (Supplementary Fig. 1), realizing the release of water within 10 min to achieve 14 sorption-desorption cycles at 15% RH and 24 cycles at 30% RH per day. In addition, the aggregation of salt particles in SHPFs is effectively suppressed, which warrants the stable water sorption performance during cycles, offering daily water yield up to 5.8 L kg^−1^ (15% RH) and 13.3 L kg^−1^ (30% RH). With all these merits, SHPFs are anticipated to accelerate the practical implementation of AWH for providing access to safely managed drinking water in a sustainable means (Fig. 1d and Supplementary Table 1).

## Results

### Preparation and characterization of SHPFs

**Fig. 2:**
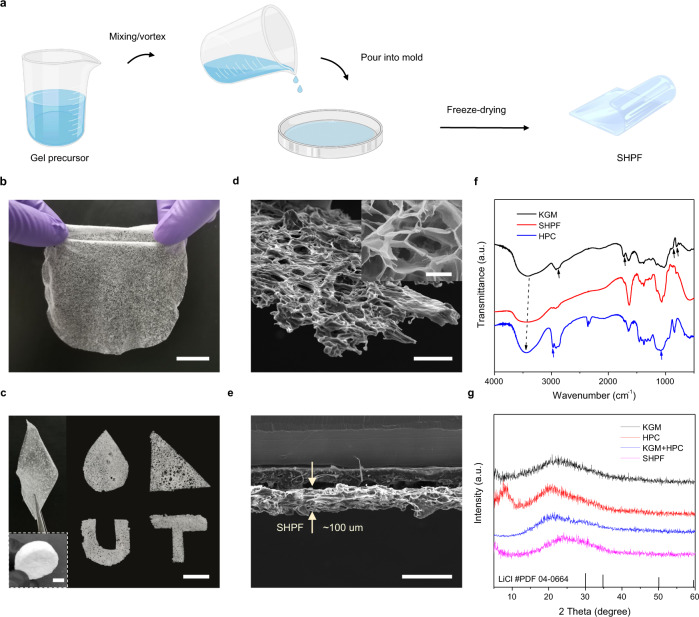
Fabrication and characterization of SHPFs. **a** Fabrication of an SHPF via a casting method. **b** Photograph of a wafer-scale SHPF. Scale bar: 2 cm. **c** Photographs of SHPFs with different shapes and thicknesses. Scale bars: 1 cm. **d** SEM image of SHPF. Scale bar: 200 µm. The Inset shows the enlarged porous structure of SHPH. Scale bar: 20 µm. **e** Cross-sectional view of SHPF showing an average thickness of ~100 µm. Scale bar: 200 µm. **f** FTIR spectra of KGM (black curve), HPC (blue curve), and SHPF (red curve). **g** The XRD patterns of KGM (black curve), HPC (red curve), KGM + HPC (blue curve), and SHPF (magenta curve). Panel **a** was partly generated using Servier Medical Art, provided by Servier, licensed under a Creative Commons Attribution 3.0 unported license.

SHPFs are synthesized via a facile, user-friendly casting method (Fig. 2a, also see Methods for more details). In a typical synthesis, a gel precursor containing KGM, HPC, and LiCl is well mixed and then poured into a mold, where the gelation takes place within 2 min without any chemical crosslinkers or initiators. This gelation of KGM/HPC gels is achieved by self-agglomeration through hydrogen bonding^23,24^. After freeze-drying, the SHPF can be peeled off from the mold for direct use. SHPFs can be easily scaled up (Fig. 2b) with tuneable sizes and shapes (Fig. 2c). From the scanning electron microscope (SEM) images, a rough surface with micro-sized pores ranging from 20–50 µm can be observed (Fig. 2d). This microporous structure is primarily facilitated by the hydrophilic KGM^25^, which enlarges the interfacial area of the SHPF and air. In addition, HPC also helps form sub-millimeter pores that are beneficial for rapid water vapor transport (Supplementary Fig. 2). To optimize the moisture sorption kinetics (Supplementary Figs. 3–5), the SHPF with a thickness of ~100 µm is used for AWH experiments in this work (Fig. 2e).

The chemical composition of SHPFs is analyzed by Fourier transform infrared (FTIR) spectroscopy (Fig. 2f). In the spectrum of KGM (black curve), the absorption bands at 3390 and 2876 cm^−1^ are assigned to the stretching of –OH groups and C–H of methyl, while the characteristic peaks of mannose appear at 875 and 804 cm^−1^ ^26,27^. The peaks of HPC (blue curve) at 1060 and 2971 cm^−1^ are assigned to the –CH_2_–O–CH_2_ stretching and the –CH stretching of the methyl group, respectively^28^. The spectrum of SHPF (red curve) contains the combination of all characteristic peaks of KGM and HPC, confirming their co-existence in the SHPF network. Meanwhile, a clear shift of –OH stretching (3428 cm^−1^) indicates the formation of a hydrogen bond between the –OH groups of HPC (3452 cm^−1^) and the –OH groups of KGM (3390 cm^−1^)^27^.

X-ray diffraction (XRD) is used to evaluate the crystallinity of SHPFs (Fig. 2g). The XRD pattern of pure KGM has a broad band at 20° due to its amorphous nature (black curve)^29^. Two characteristic diffraction peaks of HPC (red curve) are recognized at 2$\theta$ = 9° and 20°, which are attributed to the crystalline phase and amorphous phase of HPC^30^, respectively. The diffraction peaks become flatter and broader in the blend film (blue curve), indicating the good miscibility of KGM and HPC^31^. Note that the crystalline phase of HPC at 2*θ* = 9° does not appear in KGM and HPC blend film because the amount of integrated HPC is low (1.0 wt%). After the integration of LiCl into the KGM + HPC gel network, no crystalline LiCl is detected (magenta curve) due to its relatively low concentration in the polymer matrix (Supplementary Figs. 6 and 8). Since deliquescent salt powders (e.g., LiCl, CaCl_2_) will agglomerate during the hydration that can deteriorate the performance^10^, this uniform distribution of LiCl in SHPFs is favorable for sorbent materials to achieve stable functionality. With the highly porous structure and excellent capability to hold LiCl and avoid the aggregation of salt particles, SHPFs are highly desired in AWH technologies.

### AWH performance of SHPFs

**Fig. 3:**
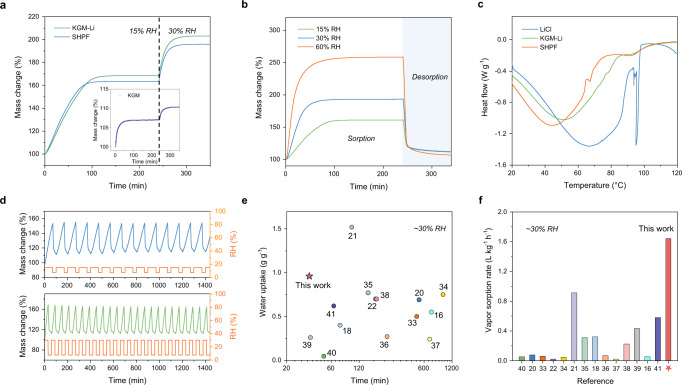
Water vapor sorption-desorption performance of SHPFs. **a** Dynamic water vapor sorption process of KGM-Li, SHPF, and pure KGM (inset) at 25 °C, 15% RH and 30% RH. **b** Dynamic water vapor sorption-desorption processes of the SHPF at different conditions. **c** Evaporation behaviors of hydrated LiCl, KGM-Li film, and SHPF. **d** Cycling performance of SHPFs, where sorption occurs at 15% RH (top: 100 min per cycle, 14 cycles per day) and 30% RH (bottom: 60 min per cycle, 24 cycles per day). The desorption is performed at 7.5% RH, 60 °C. **e** Comparison of water uptake and time required to reach 80% of the saturated water uptake with the state-of-the-art sorbent materials at ~30% RH. **f** Comparison of vapor sorption rate with the state-of-the-art sorbent materials at ∼30% RH.

The water vapor sorption is tested with a dynamic vapor sorption (DVS) system under constant humidified airflow. Water vapor from the humid air diffuses through the open pores (Fig. 2b) of SHPF to its wall due to the reduced vapor pressure at the hydrogel–air interface caused by the hygroscopic LiCl^32^. The sorption of water vapor occurs at the liquid–gas interface inside the SHPF. Then, the absorbed water quickly diffuses to the polymer network (KGM and HPC), leading to a volumetric expansion of the SHPF. The initial sorption tests on the pure KGM film show the rapid saturation of water uptake within 20 min under low RH (≤30% RH) due to its highly porous network (inset of Fig. 3a), which sets a good basis as the sorbent framework. The addition of LiCl (7.3 wt% Li^+^) with high affinity to water vapor boosts the overall water uptake to 0.69 g g^−1^ at 15% RH and 1.03 g g^−1^ at 30% RH (Fig. 3a). It should be noted that further increasing the LiCl content in SHPFs could elevate the water uptake, but the sorption kinetics became much slower (Supplementary Fig. 7). In addition, introducing HPC in SHPFs only slightly lowers the water uptake but notably enhances the water release process, which will be discussed later. The measured water uptake of the optimized SHPF is 0.64 g g^−1^ at 15% RH, 0.96 g g^−1^ at 30% RH, and 1.53 g g^−1^ at 60% RH (Fig. 3b). The sorption time required to reach 80% of its saturated water uptake at 15, 30, and 60% RH is 67, 36, and 28 min, respectively, which outperforms most of the state-of-the-art sorbent-based AWH materials (Fig. 3e)^16,18,20–22,33–41^. The calculated vapor sorption rate of the SHPF is 1.65 L kg^–1^ h^–1^ at 30% RH, which indicates 1 kg of SHPF can capture 1.65 L of water vapor in 1 h, compared favorably among the state-of-the-art sorbent materials (Fig. 3f)^16,18,20–22,33–41^.

The captured water in the SHPF can be released by >70% within 10 min through mild heating at 60 °C under a wide range of RH (Fig. 3b and Supplementary Fig. 15). This mild heating temperature is realized by introducing the hydrophilic thermoresponsive HPC with optimized molecular weight, concentration, and pH of the initial precursor (Supplementary Figs. 9–14 and Supplementary Table 2)^42^. We further measured the evaporation behaviors of pure LiCl, KGM-Li film, and SHPF via differential scanning calorimetry (DSC). Compared to the evaporation peak of KGM-Li film at 53 °C, the evaporation peak of SHPF shifted to 44 °C (Fig. 3c), which is consistent with the phase transition temperature of SHPF (Supplementary Fig. 16), indicating that a large portion of water in SHPF starts to evaporate at a lower temperature. In principle, the breakage of hydrogen bonds between HPC and water molecules occurs at ~45 °C. However, practically, a complete transformation of HPC happens at a higher temperature^43^. As such, the desorption condition is set to be 60 °C in a closed chamber under atmospheric pressure. Furthermore, the SHPF presented a stable performance for 14 cycles per day at 15% RH (70 min capturing and 30 min releasing) and 24 cycles per day at 30% RH (30 min capturing and 30 min releasing), respectively (Fig. 3d).

**Fig. 4:**
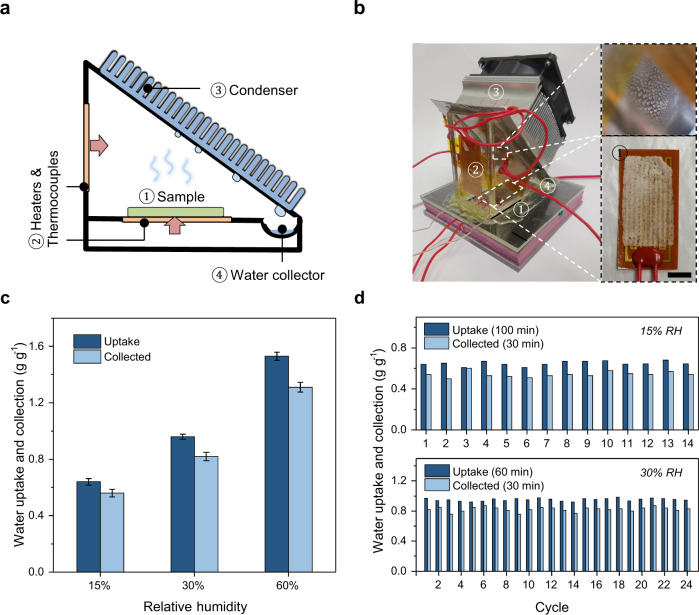
Water collection and stable cycling performance of SHPF. **a** Design scheme and **b** a photograph of the water collection device. Inset: photographs of condensed water droplets (top) and zoom-in sample (bottom). Scale bar: 1 cm. **c** Water uptake and collected water at different relative humidity. **d** Cycling performance of SHPF at 15% and 30% RH.

A water collection system is designed to further validate the ability of SHPFs to deliver freshwater from arid conditions (Fig. 4a). First, a centimeter-scale SHPF (25 mm × 40 mm) is located on top of a flexible heating plate, of which the temperature can be controlled by an external power supply (Fig. 4b). The condenser wall is tilted at ~45° for fast transport of condensed water droplets downward the water collection channel (Fig. 4b). Vertical heating plates are attached to other walls to slightly elevate their temperatures, so that water vapor can only be condensed at the cooler condensation surface to improve the water collection efficiency. It is worth noting that practically, the vertical heating plates are usually not necessary when the environmental temperature is low enough for vapor condensation. Thermocouples are attached to the backside of heating plates and condenser surface to monitor and control the temperature. This device is properly sealed with a rubber ring and steel binder clips during water collection experiments. The moisture capturing via the large SHPF is done in a homemade chamber at targeted RH prior to each water collection test (Supplementary Fig. 3). The average water collection of SHPF is 0.56 g g^−1^ after it captured moisture at ~15% RH, 0.82 g g^−1^ for ~30% RH, and 1.31 g g^−1^ for ~60% RH (Fig. 4c). The calculated average water collection efficiency (i.e., the ratio of water collection to water uptake) reaches 87%. A continuous cycling test of SHPF shows an average water collection at 0.53 g g^−1^ (~15% RH) and 0.83 g g^–1^ (~30% RH) with low Li^+^ residues (Fig. 4d and Supplementary Fig. 17), corresponding to 5.8 L kg^−1^ and 13.3 L kg^–1^ per day, which highlights the attractive water delivery capability of SHPF. Featured with high water uptake under low RHs at outdoor conditions (~5.5 L kg^−1^ day^−1^ at 10.6–41.6% RH, estimated ~12 mL_water_ kg_device_^−1^, Supplementary Fig. 18), low cost of materials (Supplementary Table 3 and Supplementary Fig. 19), facile and scalable synthesis, and overall environmental friendliness, SHPFs hold promise for future sustainable implementation.

## Discussion

We develop SHPFs as a new class of sorbent materials to harvest atmospheric water from arid climates (RH < 30%). The polymer matrix of SHPFs consists of scalable, inexpensive, non-toxic biomasses (KGM and HPC), which facilitate the hierarchically porous structure for enhanced sorption-desorption kinetics. In addition, the KGM/HPC network suppresses the aggregation of hygroscopic salt particles during the hydration process to enable remarkable water uptake with stable cycling performance. The captured water in SHPFs can be rapidly released by electric heating-assisted hydrophilic-hydrophobic switching of HPC chains, leading to 14–24 cycles of operation with a water yield of up to 13.3 L kg^–1^ per day. Considering the thin thickness of SHPF, scaling SHPF into multi-layered sorbent beds or vertical sorbent arrays is expected to further increase the water production in a unit area with improved compactness. It is anticipated that SHPFs offer possibilities to fulfill the technological gap for the development of cost-effective and sustainable AWH systems, which will be critically demanded in many arid areas as well as water-stressed communities.

## Methods

### Chemicals and materials

HPC (average MW: ~80,000, 370,000 and 1000,000), sodium hydroxide (NaOH, ACS reagent), hydrochloric acid (HCl, ACS reagent) and lithium chloride (LiCl, 99%) were purchased from Sigma-Aldrich. Low-grade KGM was purchased from Modernist Pantry^®^ on Amazon. All chemicals were used without further purification.

### Fabrication procedures

In a typical fabrication, LiCl powder (0.32–0.82 g) is added into 10 mL HPC solution (0–2.0 wt%) forming solution A. The pH of solution A can be tuned by NaOH or HCl solution. 0.44 g KGM powder is added into solution A and quickly cast into the petri dish after vortex. The gelation takes place within 2 min, and sit in room temperature for 15 min. Then, the film is placed in the fridge (−4 °C) for 3 h followed by 15 min freeze in liquid nitrogen. Last, the gel film is ready to use after 12 h freeze-drying. The final LiCl concentration in SHPFs is characterized by TGA.

### Characterizations

The SEM images were taken by FEI Quanta 650 ESEM to observe the morphology and microstructure of samples. The SHPF samples were freeze-dried for 24 h before taking SEM images. The FTIR spectra were conducted by the FTIR Spectrometer (Thermo Mattson, Infinity Gold FTIR) equipped with liquid nitrogen cooled narrow band mercury cadmium telluride detector, using an attenuated total reflection cell equipped with a Ge crystal. The TG analysis was conducted by the thermogravimetric analyzer (PerkinElmer, TGA 4000) with an airflow rate of 25 mL min^−1^ and a heating rate of 10 °C min^−1^. The sorption-desorption performance was measured by a DVS system (Surface Measurement Systems LTD, DVS Adventure). Samples are preheated at 90 °C, 0% RH for 60 min in the testing chamber for completed drying, and then placed at 25°C for 30 min stabilization. The desorption process is conducted at 60 °C, 4.5−20% RH. The airflow of DVS is fixed at 200 mL min^–1^ for all samples as the standard setting. Since the diameter of the sample holder is 0.8 cm, for each measurement, three pieces of SHPF with a diameter of 0.7 cm are loaded to fully occupy the sample holder. The evaporation behaviors of gel films (open Al crucible) and their phase transition behaviors (hermetic Al crucible) are evaluated by a differential scanning calorimeter (TA instrument, DSC 250). The scan rate of the DSC test is fixed at 2 °C min^–1^ for all samples. XRD profiles were obtained by an X-ray Diffractometer (Rigaku, Miniflex 600). The concentration of ions was tracked by inductively coupled plasma mass spectrometry (ICP-MS, Agilent 7500ce).

### Water vapor sorption measurement

Before the water vapor sorption measurement, all samples are dried in a vacuum oven at 90 °C for at least 2 h. Completely dried SHPF samples were set in a homemade RH-controlled system (Supplementary Fig. 3). The RH in the testing chamber was stabilized to the desired value by a certain super-saturated solution of a specific salt. The RH of ~15% and ~30% were realized by the super-saturated solution of LiCl and CH_3_CO_2_K, respectively, with a 250 mL min^–1^ dry airflow rate^44^. A hygrometer was employed to monitor the RH and temperature in the chamber. Then the weight change was obtained by microbalance. Commercial dehumidifier was used to assist the environmental RH during the testing and measuring steps. For cycling test with water collection measurement, the weight of samples is measured together with the sample holder to reduce experimental errors. To measure the evaporated water accurately, we use a pipette to carefully extract all the collected water and weigh it within a vial (the total mass deducts the mass of the empty vial).

## Supplementary information

Supplementary information

## Data Availability

All the data needed to evaluate the conclusions in the paper are present in the paper and/or the Supplementary information.
