# Supplementary material for: Scalable super hygroscopic polymer films for sustainable moisture harvesting in arid environments
Source: Nat Commun. 2022 May 19;13:2761. doi: 10.1038/s41467-022-30505-2 (PMC9120194; doi:10.1038/s41467-022-30505-2)
Supplement: Supplementary file 1 — Supplementary information [file 41467_2022_30505_MOESM1_ESM.pdf]

Supplementary Information

Scalable super hygroscopic polymer films for sustainable moisture harvesting in arid environments

Youhong Guo[1,2], *Weixin Guan*[1,2], *Chuxin Lei*[1], *Hengyi Lu*[1], *Wen Shi*[1], *Guihua Yu*[1,*]

[1]Materials Science and Engineering Program and Walker Department of Mechanical Engineering, The University of Texas at Austin, Austin, TX 78712, USA.

[2]These authors contributed equally to this work.

[*]Email: ghyu@austin.utexas.edu (G.Y.)

Schematic of phase transition of HPC

At low temperatures (<45 °C), the hydrophilic hydroxyl groups (-OH) on HPC attract water molecules via hydrogen bonding. While increasing the temperature above 45 °C, the hydrogen bonds are dissociated and $-CH_3$ groups on HPC facilitate hydrophobic interactions, which is known as the hydrophilicity-to-hydrophobicity transition of HPC[1, 2] (Supplementary Fig. 1). This phase transition behaviour is explored to assist the water release from SHPF.

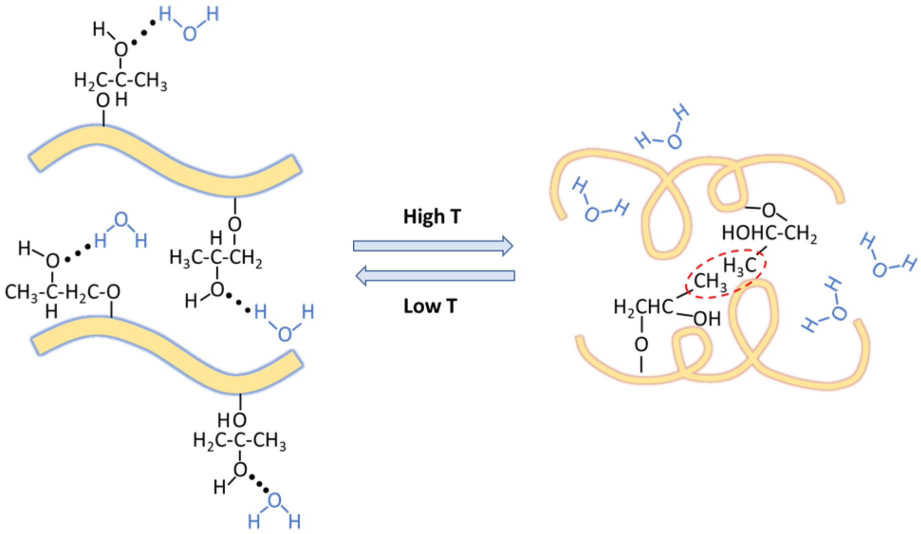

Supplementary Fig. 1. Schematic of the hydrophilicity-to-hydrophobicity transition of HPC driven by the hydrophobic interactions of $-CH_3$ groups.

Comparison of different materials for AWH

We provide the qualitative comparison of typical materials for AWH in terms of water uptake, kinetics, costs, scalability, and sustainability, which are the key parameters for practical applications (Fig. 1d and Supplementary Table 1). According to United Nations, sustainability means meeting our own needs without compromising the ability of future generations to meet their own needs[3]. Sustainable materials are materials used throughout the consumer and industrial economy that can be produced in required volumes without depleting non-renewable resources and without disrupting the established steady-state equilibrium of the environment and key natural resource systems[4]. Specifically, sustainability refers to the toxicity, renewability, and eco-friendliness of materials since those factors are equally important related to environmental sustainability and human health. Zeolites are a class of materials that have been widely applied in the industry for separation and catalysis. They possess the merits of mature techniques of both materials production and device integrations. However, the cost and AWH performance of zeolites need to be improved[5-7]. Hygroscopic salts demonstrate good water uptake; however, they show sluggish kinetics due to the passivation layer formed in the deliquescence process, which undermines their potential for practical applications[8]. MOFs have emerged as a promising sorbent platform for AWH, especially in arid environments. It has already presented great potential for device integration with stable AWH performance and relatively low costs[9-13]. Still, intensive ongoing efforts are invested in the green and facile synthesis of MOFs towards environmental sustainability[10, 14]. Our SHPF demonstrates comparable AWH performance in arid conditions. The raw materials (KGM and HPC) are low-cost and renewable biomasses, which show clear environmental and economic advantages (Supplementary Table 3). In addition, the fabrication of SHPF does not involve high energy consumption (e.g., high temperature, high pressure), expensive,

complicated equipment, nor environmentally unfriendly chemicals (see Methods: Chemicals and materials; Fabrication procedures). With this simple fabrication process, the size of SHPF can be easily scaled up or changed as we demonstrated (Fig. 2b, 2c, and Supplementary Fig. 18a)).

Supplementary Table 1. Evolution matrices of the cross-comparisons in terms of water uptake, kinetics, and costs.

**Score**	water uptake at 30% RH (g g^{-1})
1	<0.4
2	0.4-0.6
3	0.6-0.8
4	0.8-1.0
5	>1.0
**Score**	Time to reach 80% water uptake (min)
1	>200
2	150-200
3	100-150
4	50-100
5	<50
**Score**	cost ($\$ \text{ kg}^{-1}$)
1	>60
2	40-60
3	20-40
4	10-20
5	<10

Surface morphology of dehydrated SHPFs

Hydrophilic KGM facilitates micropores, and HPC helps form interconnected pores as well as sub-millimetre pores for water vapor transport (Supplementary Fig. 2). Such a surface morphology greatly enlarges the exposure area to the humid air, resulting in rapid sorption kinetics.

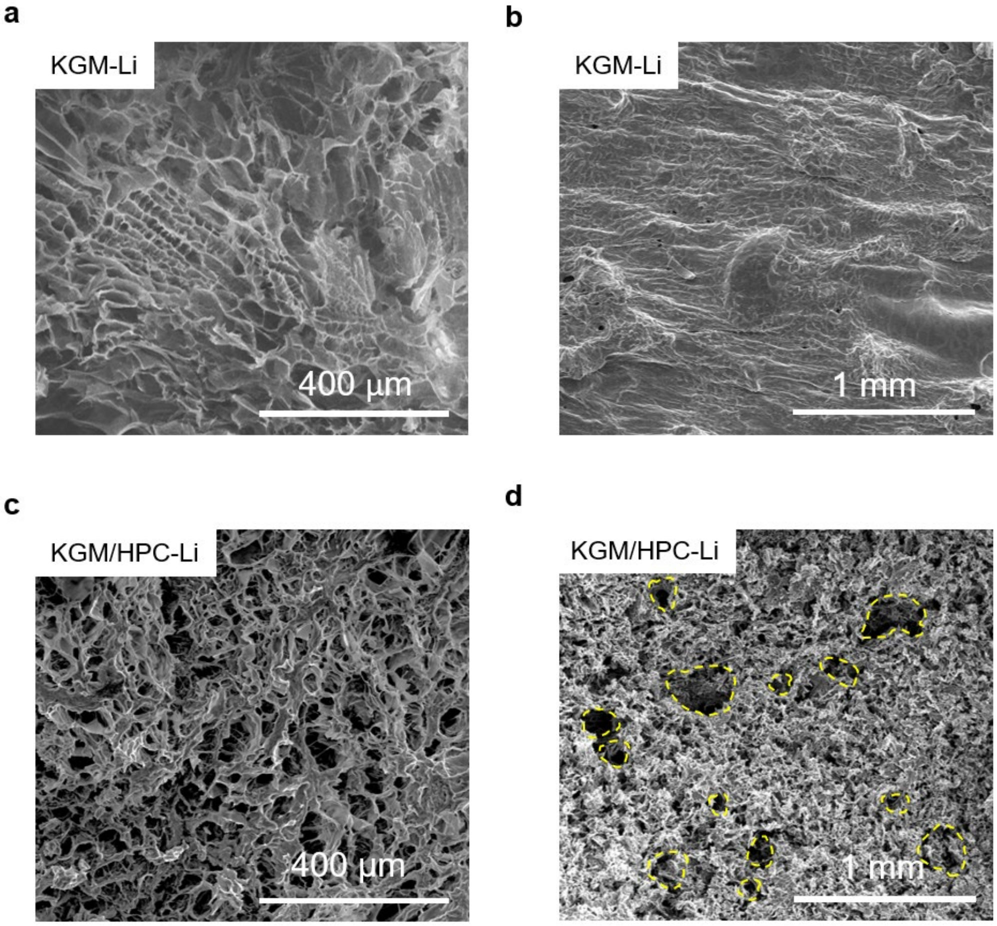

Supplementary Fig. 2. **SEM images of dehydrated gels. a**, **b** KGM-Li. **c**, **d** KGM/HPC-Li. Dashed circles: sub-millimetre pores.

Homemade water vapor sorption measurement setup

Briefly, the homemade vapor sorption system consists of three parts (see Methods): RH controller, testing chamber, and a hygrometer (Supplementary Fig. 3). Supersaturated salt solutions are applied to generate airflows with controlled RHs by supplying dehydrated airflow into solutions. Typically, supersaturated LiCl and CH_3CO_2K solutions are used for ~ 15% and 30% RH conditions. The testing chamber is sealed by a rubber ring. The hygrometer is inserted to monitor the RH and temperature in the sorption chamber.

a

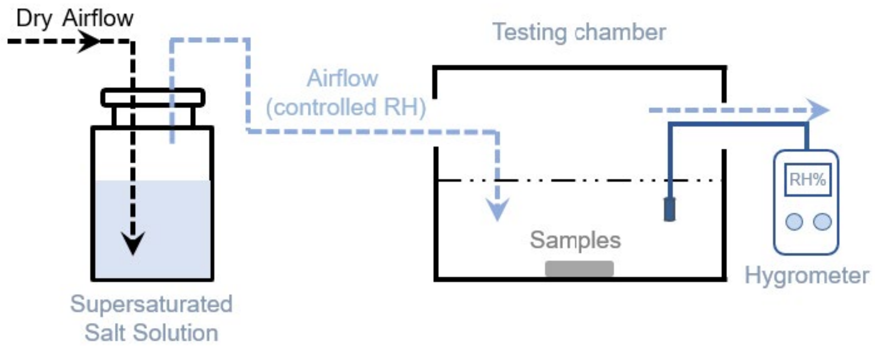

b

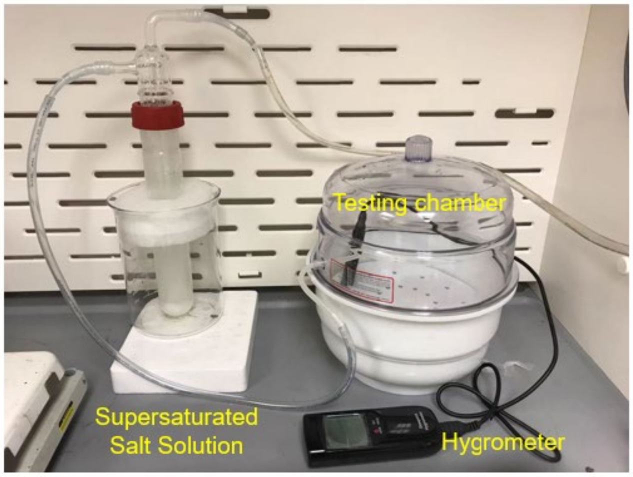

Supplementary Fig. 3. **Water vapor sorption measurement setup**. (**a**) Schematic and (**b**) photograph of the homemade water vapor sorption system.

Tuning the concentration of KGM

The effect of KGM contents on water uptake was investigated (Supplementary Fig. 4). The KGM concentration is optimized to be ~4.5 wt% with the highest water uptake. The water uptake decreases with continuously increasing KGM content to ~9 wt%. To understand this result, we note that the gelation of KGM hydrogel is achieved by self-agglomeration through hydrogen bonding so that increasing the concentration of KGM will lead to a higher physical crosslinked network (i.e., denser network). It has been theoretically demonstrated that reducing cross-linking can lead to higher water volume fraction within hydrogel, which means more water from the air can be trapped[15]. A loosed network can be formulated by lowering the KGM concentration to achieve higher water uptake. However, it should be noted that the continuing decrease of the concentration of KGM to ~2.0 wt% cannot result in the formation of hydrogels with a decent mechanical property[16].

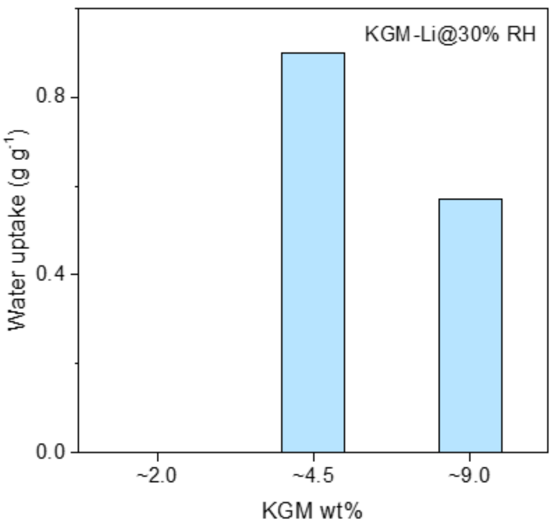

Supplementary Fig. 4. Water uptake of KGM-Li gel with different KGM concentrations.

Thickness dependence of water uptake

By adjusting the volume of casting solutions, SHPFs with different thicknesses can be fabricated. Typically, SHPFs with the thickness of ~100 μm, ~160 μm, and ~250 μm were prepared by casting 4 mL, 6 mL, and 8 mL precursor solutions, respectively. The moisture sorption process demonstrates thickness-dependent kinetics based on the DVS results (Supplementary Fig. 5). The SHPF with the lowest thickness (~100 μm) exhibits the best water uptake amount (0.64 g g^{-1} at 15% RH and 0.96 g g^{-1} at 30% RH) and the fastest kinetics. It can reach 80% of saturated water uptake in 60 min and 20 min at 15% RH and 30% RH, respectively. In comparison, SHPF with a moderate thickness (~160 μm) can harvest a similar amount of moisture with a slightly longer time. The SHPF with a thickness of ~250 μm presents the sluggish kinetics, failing to reach the equilibrium plateau under the same time scale. The kinetics of hydrogel-salt composite is highly affected by its thickness because the resistance of both vapor and liquid transport decreases with decreased thickness[17-19]. It should be noted that continuingly decreasing the thickness below 100 μm could potentially increase the kinetics further, but considering the pore size (20-50 μm), the porous film with a thickness below 100 μm may not be able to maintain its mechanical structure.

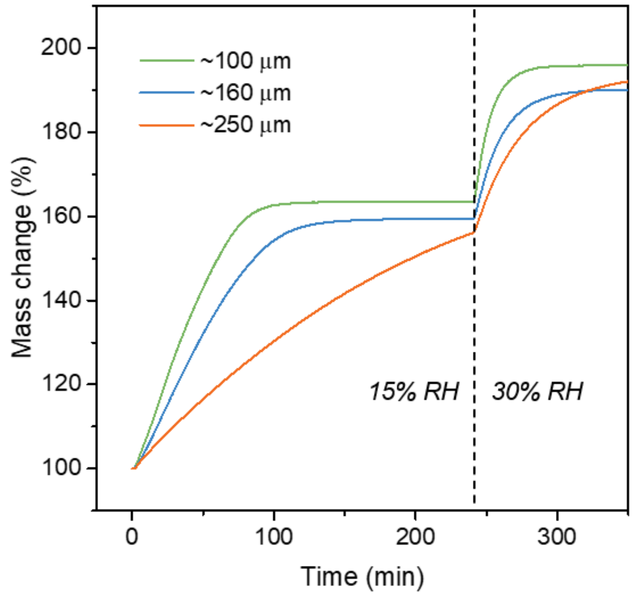

Supplementary Fig. 5. Dynamic water vapor sorption of SHPFs with different thicknesses.

Chemical compositions of SHPFs

A thermogravimetric analyzer (TGA) was adopted to evaluate the chemical composition of SHPF and KGM+HPC film samples after pre-drying under 100 °C for 24 hours (Supplementary Fig. 6). From 30 to 100°C temperature, all samples showed initial weight loss due to the escape of physically adsorbed water. For KGM+HPC film, the weight loss around 250°C to 350°C corresponded to the depolymerization of KGM chains and thermal scission of chemical bonds accompanying the dehydration of saccharide units, as well as the ring-opening polymerization of HPC[20, 21]. The second weight loss above 350°C is attributed to the further oxidation of each derivative[22]. The polymers decomposed completely after 500°C. The fully hydrated SHPF contains 90 wt% of water (blue curve). The water evaporated below 100°C and then the polymer matrix gradually decomposed from 100-600°C. The remaining weight (~6 wt%) was attributed to the weight percentage of LiCl in the hydrated SHPF, which is in agreement with the theoretical concentration of LiCl in SHPF hydrogel. In comparison, the main weight loss of dehydrated SHPF begins at 200°C, slightly lower than that of KGM+HPC, which is attributed to the coupling of polymer decomposition and salt-bonded water[23, 24]. Dehydrated SHPF remains around ~44 wt% residues when the temperature reaches 600°C.

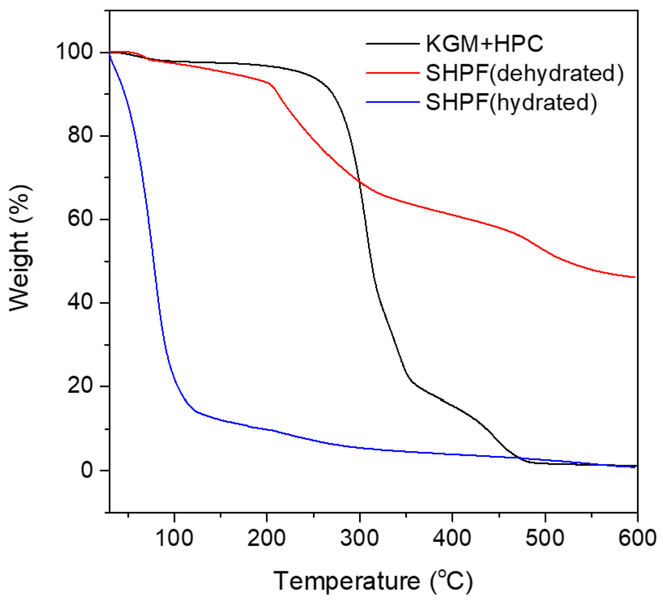

Supplementary Fig. 6. TGA curves for the KGM+HPC film, SHPF hydrogel, and dried SHPF.

Tuning the salt concentration in SHPFs

Although hygroscopic salts are proved to be effective for moisture capture, the slow kinetics and high energy consumption for the regeneration remain problematic[19, 25]. Therefore, there are many attempts to integrate salts into different matrices, such as MOFs[26] and aerogels[19]. Among these candidate materials, hydrogels stand out due to their intrinsic hydrophilicity and facile fabrication[27, 28]. Here, in SHPFs, the salt concentration was tuned to achieve higher water uptake as well as to optimize the kinetics since increasing salt content can boost the sorption capacity but slower the kinetics. Increasing salt content from 3.7 wt% to 7.3 wt% Li^+ shows an increased water sorption capacity with similar kinetics at 15-30 % RH. However, continue increasing salt content to 10.9 wt% Li^+ leads to much slower kinetics. Both sorption profiles of pure LiCl and the SHPF with 10.9 wt% Li^+ failed to reach the saturated state within four hours (Supplementary Fig. 7). In addition, crystalline LiCl cannot be detected until the concentration of Li^+ is increased to 9.3 wt% (Supplementary Fig. 8). The sharp peaks at 2θ = 30.1°, 34.8°, 50.2°, 59.6°, and 32.5° are attributed to the planes of (1 1 1), (2 0 0), (2 2 0), (3 1 1), and (2 2 2) of LiCl crystal, which is in the good alignment of standard XRD pattern of PDF#00-004-0064. The peak at 2θ = 32.8° is attributed to the (2 0 2) plane of $LiCl \cdot H_2O$ based on the standard XRD pattern of PDF#00-022-1142, which is due to the rapid vapor sorption of the sample during the measurement[29]. In comparison, SHPFs with lower LiCl concentrations can reach equilibrium within an hour, which confirms that the highly porous structure and hydrophilic essence of the polymeric network indeed boost the kinetics. The uptake kinetics of SHPF with 3.7 wt% Li^+ (0.75 M) is slightly faster than the one with 7.3 wt% Li^+, but the water uptake is halved. As such, the optimized salt concentration is chosen to be 7.3 wt% Li^+ (1.5M LiCl).

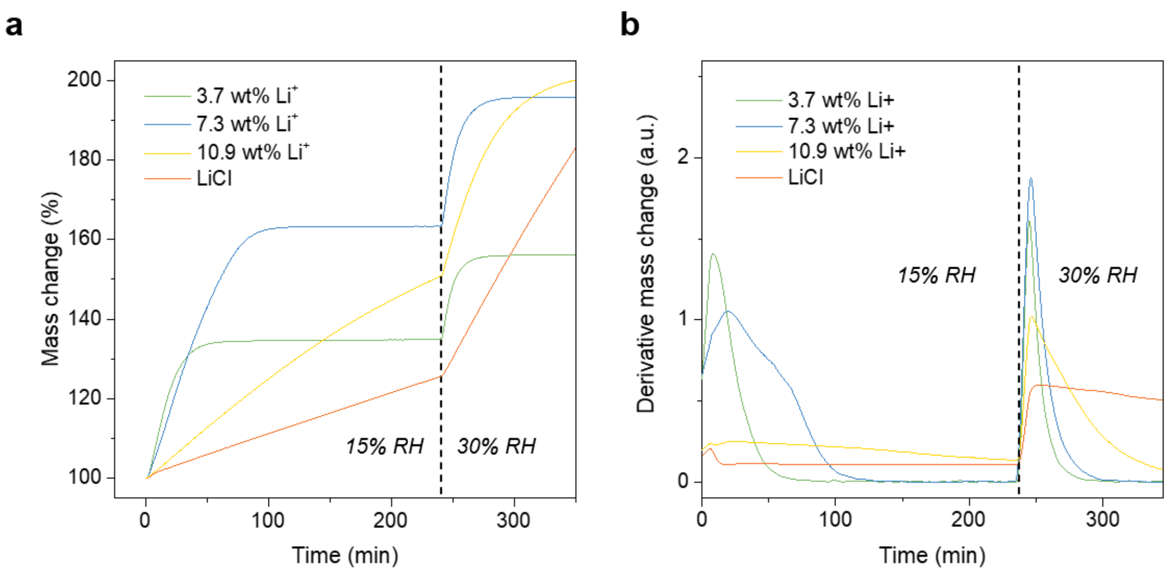

Supplementary Fig. 7. a, Water uptake of pure LiCl and SHPFs with different salt contents at 15% RH and 30% RH. **b,** Derivative mass change of pure LiCl and SHPFs with different salt contents at 15% RH and 30% RH.

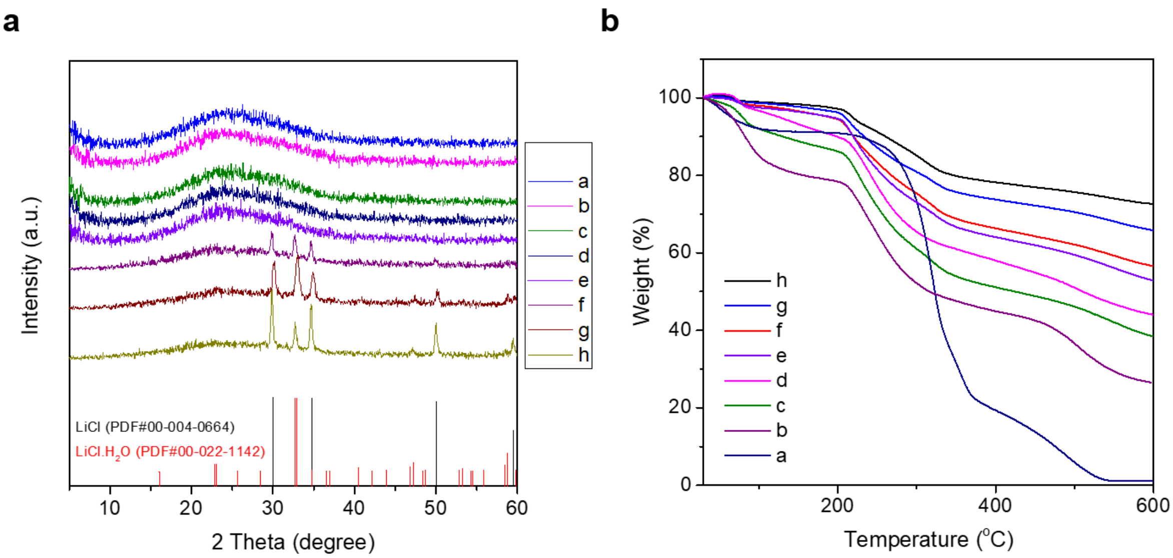

Supplementary Fig. 8. a, The XRD patterns, **b,** TGA of the SHPF with 0-12.0 wt% Li+. (a: 0 wt%; b: 3.7 wt%; c: 6.3 wt%; d: 7.3 wt%; e: 8.4 wt%; f: 9.3 wt%; g: 10.9 wt%; h: 12.0 wt%;)

Tuning the molecular weight of HPC

The molecular weight (MW) of HPC also impacts the water uptake performance (Supplementary Fig. 9). HPCs with an average MW of ~80,000, 370,000 and 1000,000 are denoted as HPC 1, HPC 2 and HPC 3, respectively. The water uptake amount and kinetics both increase with the decreasing MW of HPC because a moderately loose framework can enhance mass transfer with a highly porous internal structure. It should also be noted that HPC 3 is highly viscous at 1 wt%, which is not favourable in forming a homogenous hydrogel. In this work, HPC 1 with a MW of ~80,000 is used.

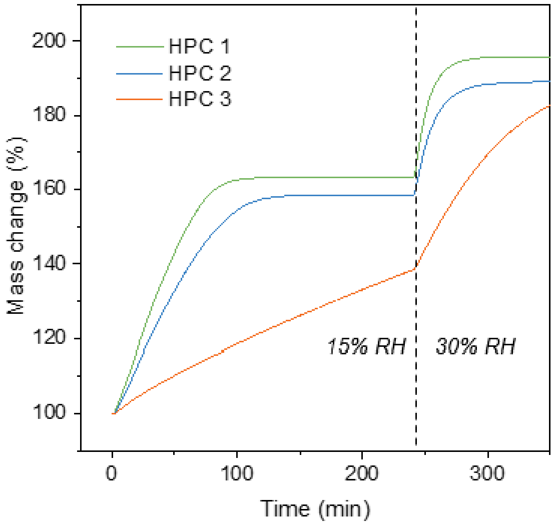

Supplementary Fig. 9. Water uptake of SHPFs with different molecular weights of HPC.

Tuning the concentration of HPC in SHPFs

The effect of HPC content on water uptake and collection was investigated. The water uptake decreases with increased HPC contents as HPC does not contribute much to the moisture capturing (Supplementary Fig. 10). HPC is integrated into SHPF mainly to facilitate water release. After increasing HPC from 0.5 wt% to 1.0 wt%, a significantly lowered evaporation peak from 50°C to 44°C was observed (Supplementary Fig. 11). However, the continuing increase of HPC concentration from 1.0 wt% to 2.0 wt% did not show a lower evaporation temperature. The KGM/HPC hydrogel with ≥1.0 wt% of HPC also showed more apparent bulk release of water upon heating (Supplementary Fig. 12). In addition, the 1.0 wt% HPC only had a slightly lower water uptake than 0.5 wt% HPC but provided a clear advantage on water collection efficiency (Supplementary Fig. 13). Thus, 1.0 wt% HPC is selected in this work for later water uptake tests as well as multi-cycle operations.

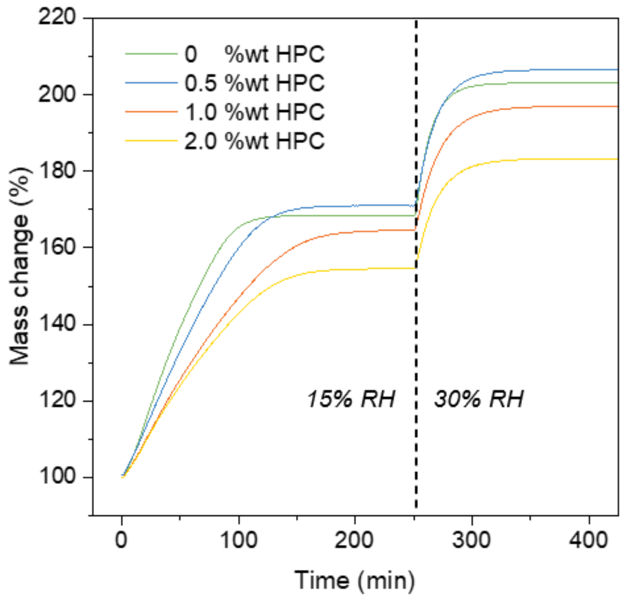

Supplementary Fig. 10. Water uptake of SHPFs with different weight percentages of HPC.

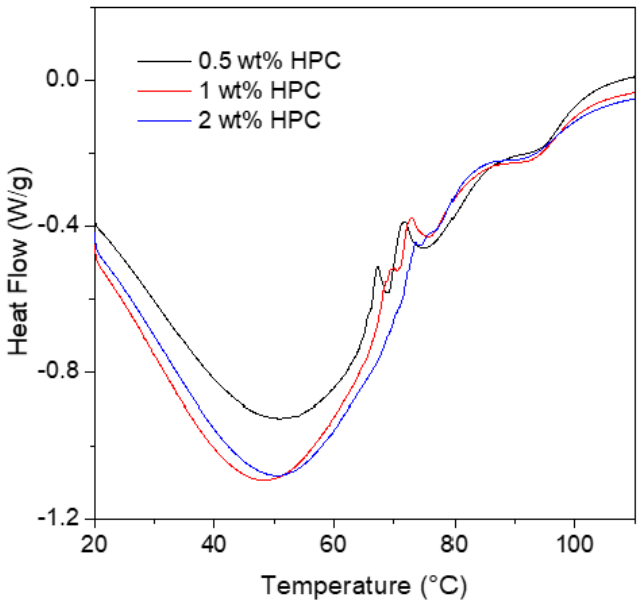

Supplementary Fig. 11. Evaporation behaviour of SHPFs with different weight percentages of HPC by DCS test.

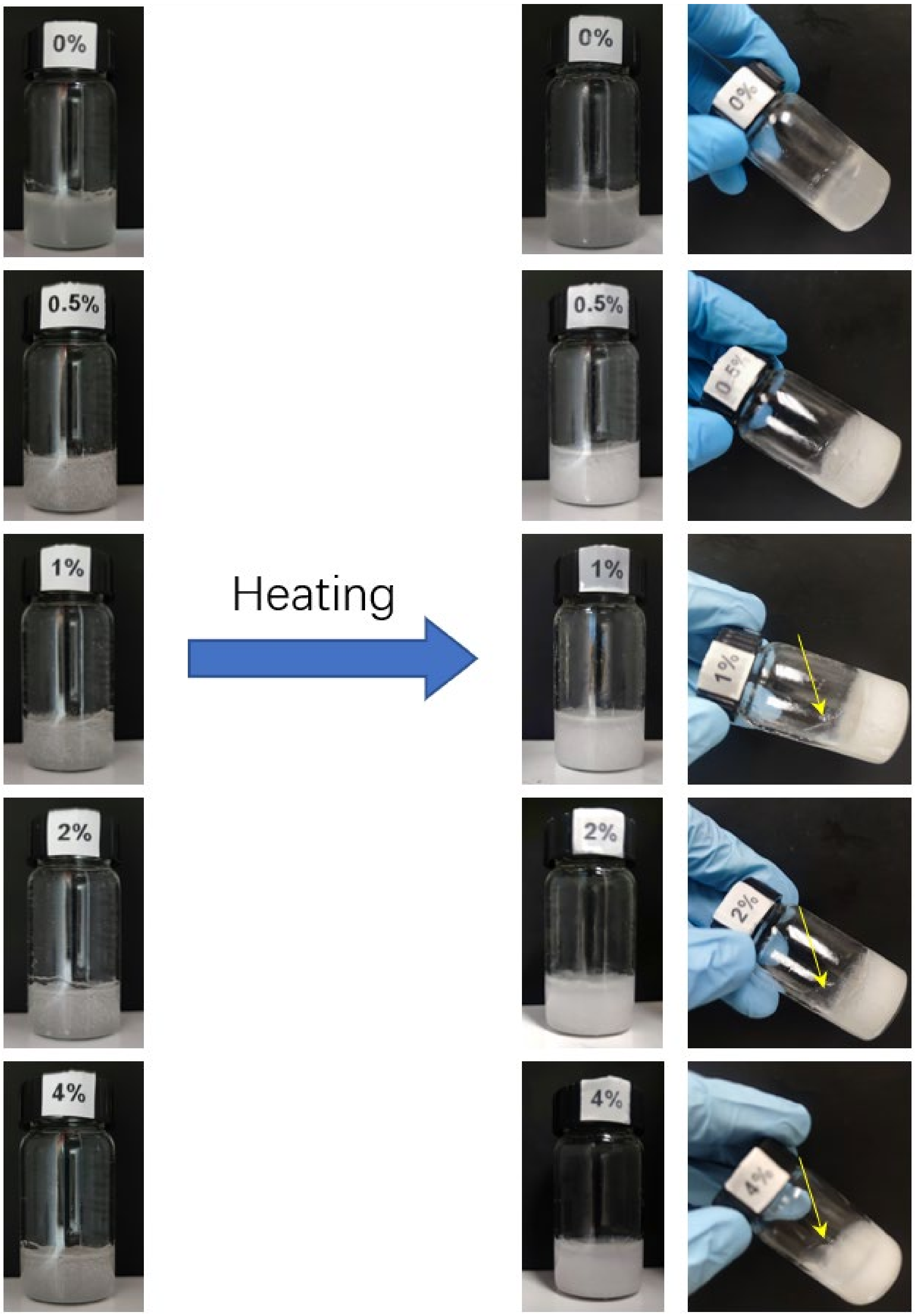

Supplementary Fig. 12. Bulk release of water from KGM/HPC hydrogel with different HPC concentrations after being heated at 60°C for 30 min.

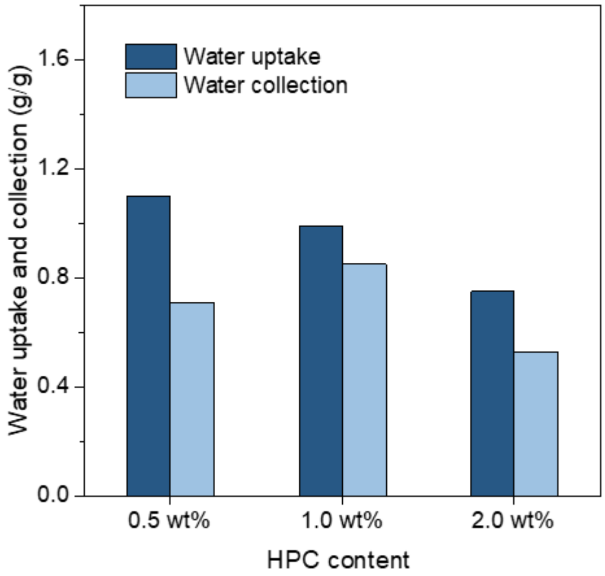

Supplementary Fig. 13. **Water uptake and collected water of SHPFs with different weight percentages of HPC by evaporation-condensation water collection test.**

pH effect of initial HPC precursor on water uptake

The transition temperature of HPC can be varied by tuning the pH of the HPC solution[30]. Typically, both acid and alkaline conditions can lower the transition temperature of HPC[31]. As such, pH 4, 7, 8.5 of initial HPC precursors were evaluated on their water uptake and kinetics (Supplementary Fig. 14). All samples were tested in DVS and DSC system and the average values were summarized in Supplementary Table 2. All SHPFs at different pH values showed similar water uptake. The water uptake kinetics of SHPF at pH=8.5 stands out, which is averagely 14 min and 41 min faster than pH=4 and pH=7 to reach 80% of total water uptake at 15% RH, respectively. For the desorption process, SHPF at pH=8.5 required 64 min and 7 min less compared to SHPF with pH=4 and 7, which can be attributed to the lower evaporation peak (~44.2°C). It is noteworthy that acid conditions will induce the degradation of KGM gradually, which negatively influences the water uptake and desorption kinetics[32]. Therefore, the optimal pH value is set at 8.5.

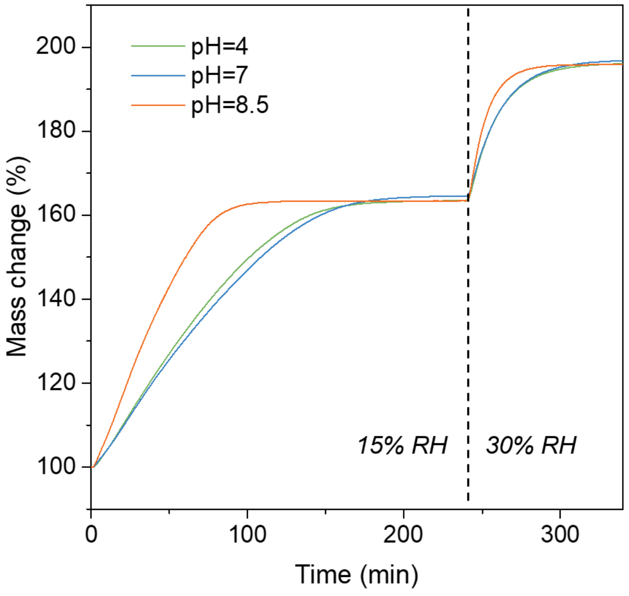

Supplementary Fig. 14. Dynamic water vapor sorption process of SHPFs with pH=4, 7 and 8.5 at 25°C, 15% RH and 30% RH.

Supplementary Table 2. Average water uptake, kinetics, and evaporation temperature peak of SHPFs with different pH values.

**pH of HPC precursor**	**4**	**7**	**8.5**
Water uptake at 15% RH (g g^{-1})	0.63	0.65	0.64
Sorption time to reach 80% water uptake at 15% RH (min)	74	101	60
Desorption time to release 70% of water uptake (min)	18	13	9
Desorption time to release 80% of water uptake (min)	100	43	36
Evaporation peak measured by DSC (ºC)	45.3	49.1	44.2

Desorption conditions of SHPFs

Different desorption conditions of SHPFs are evaluated using DVS at 15% RH and 30% RH. The samples are named based on their desorption RH and corresponding dew point. Despite the sorption RH, SHPFs are able to release ~70% of their water in 10 min under 60°C from 4.5% RH to 20% RH, which corresponds to dew point temperature from 5°C to 29°C (Supplementary Fig. 15). After 340 min, the mass change is at ~110-115%. This could be attributed to a small portion of water bound to LiCl, which requires more energy (e.g., much higher temperature, >95°C, Fig. 3c) to be released from SHPF.

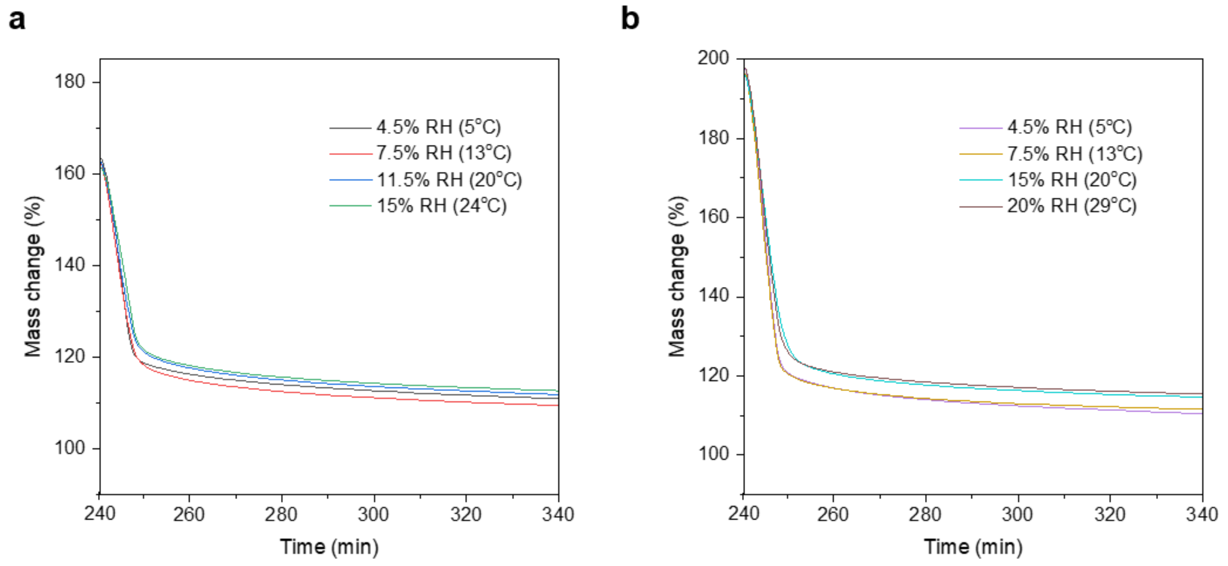

Supplementary Fig. 15. **Desorption kinetics of SHPFs at different RH. a,** After vapor sorption at 15% RH. **b,** After vapor sorption at 30% RH. The temperatures in brackets indicate the corresponding dew points.

Phase transition behaviour of SHPF

Hydroxypropyl cellulose (HPC) is integrated into SHPFs to favour the water release. DSC is used to determine the phase transition behaviour of SHPF (Supplementary Fig. 16). The samples are placed in a hermetic Al crucible and measured under nitrogen flow (50mL min^{-1}) from 20 to 65 ºC with a linear heating rate of 2 ºC/min. The heat flow signals changed with temperature. KGM-Li (red line) is a control sample where no heat signal was observed, indicating that KGM-Li does not have phase transition behaviour. In contrast, the SHPF showed a small dip between 40-50 ºC, presenting a phase transition behaviour. This is attributed to be the dehydration of SHPF driven by the hydrophobic interactions of C–H groups[1, 33]. The measured transition temperature is at 45.5 ºC of SHPF, which is consistent with the theoretical value at 45 ºC.

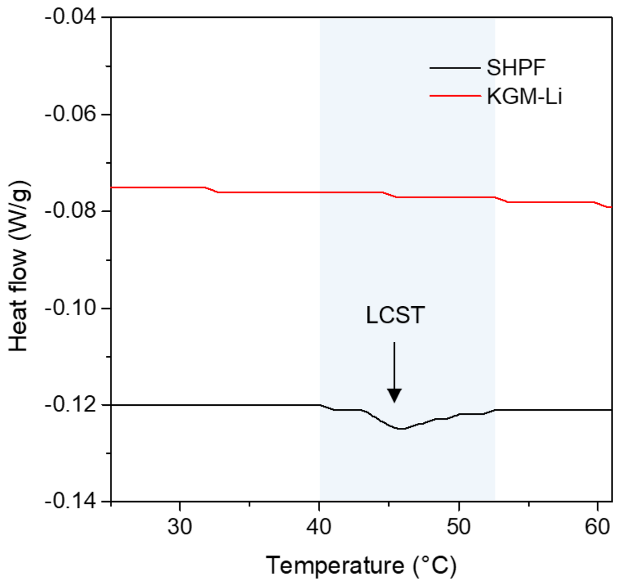

Supplementary Fig. 16. **Phase transition behaviour of the SHPF.**

Lithium residue in collected water

The KGM/HPC hydrogel presents good ability to hold and keep the LiCl within its network during the sorption-desorption process. Using electric heating enabled evaporation-condensation system (Figure 4a), the concentration of Li^+ in collected water is ~ 0.25 ± 0.03 ppm (mg L^{-1}) (Supplementary Fig. 17), which is slightly lower than other salt-polymer composite AWH materials due to the lower LiCl concentration used in SHPF[19, 34].

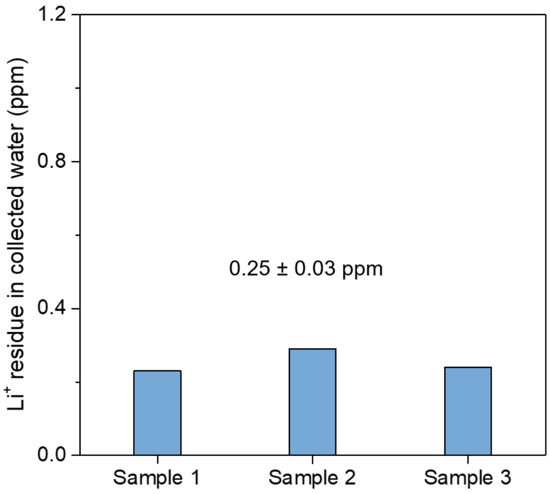

Supplementary Fig. 17. Li^+ residue in collected water samples.

Outdoor atmospheric water harvesting of SHPF

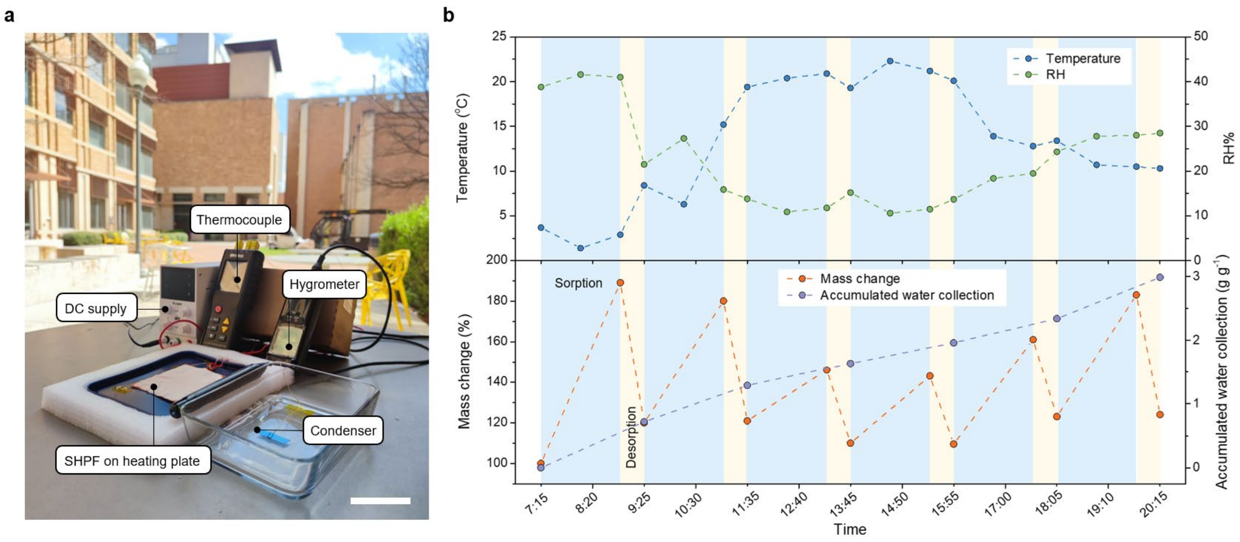

Supplementary Fig. 18. **Outdoor atmospheric water harvesting of SHPF**. **a**, A photograph of the electric heating enabled water collection device. Scale bar: 10 cm. **b,** Outdoor temperature and relative humidity (top); Mass changes of sorption/desorption processes, and accumulated water collected from condensation (bottom). The data were collected on March 12th, 2022, at Austin, Texas, USA.

Outdoor water harvesting and collection experiment based on SHPF was conducted at University of Texas, Austin campus on March 12th, 2022 (Supplementary Fig. 18a). Six cycles were performed under the RH ranging from 10.6 to 41.6% (environmental temperature of 1.4-22.3°C) to achieve a water uptake of 0.43 to 0.90 g g^{-1} (Supplementary Fig. 18b). One cycle included 100 min sorption and 30 min desorption (including condensation and collection). The power supply of the heating plate was 3.38 W for a large SHPF sample to reach and maintain at 60°C. The water vapor was condensed on the inner surface of glassware without vertical heating plates. The

condensed water was collected using a pipette. The water production is 0.3-0.7 g_{water}/g_{SHPF} per cycle, corresponding to ~5.5 L kg^{-1} day^{-1} (11 cycles per day, RH 10.6%-41.6%). The thermal efficiency calculated from each cycle is 16%-22%, which is comparable to the reported experimental thermal efficiency[35] and estimated thermal efficiency[36]. The scaling of SHPF into multilayered sorbent beds or vertical sorbent arrays is expected to further increase the energy efficiency in practical applications[5]. In addition, for next generation of SHPF, solar absorbers with simultaneous water vapor capturing function (e.g., PPy-Cl) can be integrated to boost the water uptake as well as to reduce the electricity consumption[37].

Cost analysis of raw materials and supplementary device supplies of SHPFs

The raw materials cost of the optimized SHPF was estimated to be \$1.95 kg^{-1} (Supplementary Table 3), which shows its potential for large-scale production. Biomasses used here are non-toxic, renewable, and biocompatible, which is environmentally friendly and can be used in a sustainable manner[33, 38, 39]. As a demonstration, low-grade LiCl was purchased from Amazon with a lower price compared to Sigma Aldrich and presented similar AWH performance (Supplementary Fig. 19). Based on the outdoor demonstration with a larger sample, ~4.1 kWh electricity will be needed to collect 1 L of water, corresponding to ~\$0.24 per kg of water (Texas, USA electricity price). Although specific energy consumption of a system should be highly dependent on the scale, ambient humidity and temperature, the outdoor SHPF water harvesting system shows a potential advantage over commercially available dewing systems under 20% RH (~5.6 kWh L^{-1})[7, 40]. It is worth noting that with the scaling of water harvesting device and improved engineering optimization on automatic water collection, the estimated cost of the SHPF system is anticipated to be further lowered.

Supplementary Table 3. **Raw materials cost analysis of SHPFs.**

**Raw Materials**	**Cost (\$ kg^{-1})**	**Estimated single component cost in SHPF (\$ kg^{-1})**	**Source**
**Konjac glucomannan**	1.5	0.56	https://www.alibaba.com/product-detail/Low-Price-Freshener-Konjac-Gum-powder_1600094252557.html?spm=a2700.shop_plser.41413.11.710a5129cn04oQ
**Hydroxypropyl cellulose**	3.65	0.31	https://www.alibaba.com/product-detail/Hydroxypropyl-cellulose_1858992573.html?spm=a2700.shop_plser.41413.15.6492774fcfnivI
**Lithium Chloride**	2	1.08	https://www.alibaba.com/product-detail/low-price-high-assay-cas-7447_1600443464257.html?spm=a2700.galleryofferlist.normal_offer.d_title.314a5241MgYoZz
**Total Cost (\$ kg^{-1})**		1.95	

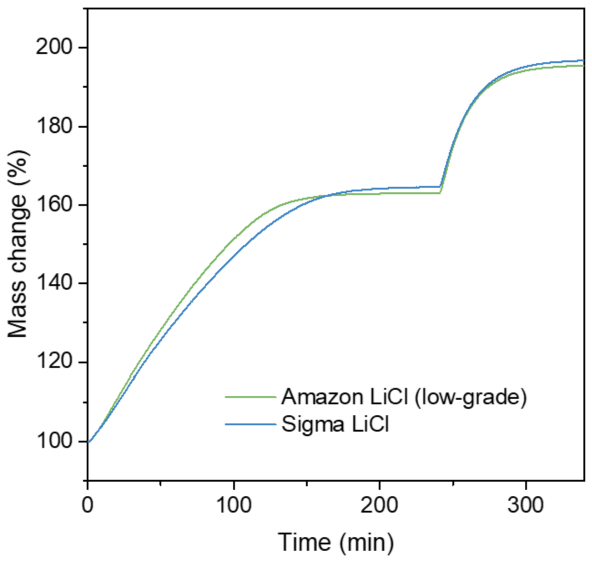

Supplementary Fig. 19. Water uptake performance of SHPF (pH=7) using different LiCl purchased from Sigma Aldrich and Amazon.

Supplementary References

1. Jing, Y. & Wu, P. Study on the thermoresponsive two phase transition processes of hydroxypropyl cellulose concentrated aqueous solution: from a microscopic perspective. *Cellulose* **20**, 67-81 (2013).
2. Gao, J., Haidar, G., Lu, X. & Hu, Z. Self-Association of Hydroxypropylcellulose in Water. *Macromolecules* **34**, 2242-2247 (2001).
3. http://www.un-documents.net/ocf-02.htm <Our Common Future, Chapter 2_ Towards Sustainable Development - A_42_427 Annex, Chapter 2 - UN Documents_ Gathering a body of global agreements.pdf>.
4. http://sustain.rutgers.edu/what_are_sustainable_materials.
5. Hanikel, N. et al. Rapid Cycling and Exceptional Yield in a Metal-Organic Framework Water Harvester. *ACS Central Sci.* **5**, 1699-1706 (2019).
6. Entezari, A., Ejeian, M. & Wang, R. Modifying water sorption properties with polymer additives for atmospheric water harvesting applications. *Appl. Therm. Eng.* **161**, 114109 (2019).
7. Lapotin, A., Kim, H., Rao, S.R. & Wang, E.N. Adsorption-Based Atmospheric Water Harvesting: Impact of Material and Component Properties on System-Level Performance. *Acc. Chem. Res.* **52**, 1588-1597 (2019).
8. Tereshchenko, A.G. Deliquescence: Hygroscopicity of Water-Soluble Crystalline Solids. *J. Pharm. Sci.* **104**, 3639-3652 (2015).
9. Fathieh, F. et al. Practical water production from desert air. *Sci. Adv.* **4**, eaat3198 (2018).
10. Hanikel, N., Prévot, M.S. & Yaghi, O.M. MOF water harvesters. *Nat. Nanotechnol.* **15**, 348-355 (2020).

11. Kalmutzki, M.J., Diercks, C.S. & Yaghi, O.M. Metal–Organic Frameworks for Water Harvesting from Air. *Adv. Mater.* **30**, 1704304 (2018).

12. Towsif Abtab, S.M. et al. Reticular Chemistry in Action: A Hydrolytically Stable MOF Capturing Twice Its Weight in Adsorbed Water. *Chem* **4**, 94-105 (2018).

13. Rieth, A.J., Yang, S., Wang, E.N. & Dincă, M. Record Atmospheric Fresh Water Capture and Heat Transfer with a Material Operating at the Water Uptake Reversibility Limit. *ACS Central Sci.* **3**, 668-672 (2017).

14. Gassensmith, J.J. et al. Strong and Reversible Binding of Carbon Dioxide in a Green Metal–Organic Framework. *J. Am. Chem. Soc.* **133**, 15312-15315 (2011).

15. Chen, G. Thermodynamics of hydrogels for applications to atmospheric water harvesting, evaporation, and desalination. Preprint at https://doi.org/10.48550/arXiv.2111.12543 (2021).

16. Zhang, C., Chen, J.-d. & Yang, F.-q. Konjac glucomannan, a promising polysaccharide for OCDDS. *Carbohydr. Polym.* **104**, 175-181 (2014).

17. Díaz-Marín, C. et al. Theory of Absorption Kinetics in Hygroscopic Hydrogels. *Bull. Am. Phys. Soc.* (2021).

18. Burnett, D.J., Garcia, A.R. & Thielmann, F. Measuring moisture sorption and diffusion kinetics on proton exchange membranes using a gravimetric vapor sorption apparatus. *J. Power Sources* **160**, 426-430 (2006).

19. Xu, J. et al. Ultrahigh solar-driven atmospheric water production enabled by scalable rapid-cycling water harvester with vertically aligned nanocomposite sorbent. *Energy Environ. Sci.* **14**, 5979-5994 (2021).

20. Wang, J. et al. Transparent konjac glucomannan/cellulose nanofibril composite films with improved mechanical properties and thermal stability. *Cellulose* **26**, 3155-3165 (2019).

21. Kowhakul, W., Shibahara, H., Masamoto, H. & Shigematsu, M. Dust explosion characteristics of cellulose ethers and cellulose acetates with various degrees of acetylation. *J. Loss Prev. Process Ind.* **44**, 544-550 (2016).

22. Kawamoto, H., Murayama, M. & Saka, S. Pyrolysis behavior of levoglucosan as an intermediate in cellulose pyrolysis: polymerization into polysaccharide as a key reaction to carbonized product formation. *J. Wood Sci.* **49**, 469-473 (2003).

23. Masset, P. Thermogravimetric study of the dehydration reaction of $LiCl \cdot H_2O$. *J. Therm. Anal. Calorim.* **96**, 439-441 (2009).

24. Dai, J. et al. LiCl loaded cross-linked polymer composites by click reaction for humidity sensing. *Sens. Actuators, B* **253**, 361-367 (2017).

25. Xu, Y., Li, L., Zheng, P., Lam, Y.C. & Hu, X. Controllable Gelation of Methylcellulose by a Salt Mixture. *Langmuir* **20**, 6134-6138 (2004).

26. Xu, J. et al. Efficient Solar-Driven Water Harvesting from Arid Air with Metal–Organic Frameworks Modified by Hygroscopic Salt. *Angew. Chem. Int. Ed.* **59**, 5202-5210 (2020).

27. Li, R., Shi, Y., Wu, M., Hong, S. & Wang, P. Photovoltaic panel cooling by atmospheric water sorption–evaporation cycle. *Nat. Sustain.* **3**, 636-643 (2020).

28. Li, R. et al. Hybrid Hydrogel with High Water Vapor Harvesting Capacity for Deployable Solar-Driven Atmospheric Water Generator. *Environ. Sci. Technol.* **52**, 11367-11377 (2018).

29. Weiss, E., Hensel, H. & Kühr, H. Röntgenographische und kernmagnetische Breitlinienresonanz-Untersuchung der Lithiumhalogenid-monohydrate. *Chemische Berichte* **102**, 632-642 (1969).

30. Lu, X., Hu, Z. & Schwartz, J. Phase Transition Behavior of Hydroxypropylcellulose under Interpolymer Complexation with Poly(acrylic acid). *Macromolecules* **35**, 9164-9168 (2002).

31. Zhang, L. et al. Energy-Saving Smart Windows with HPC/PAA Hybrid Hydrogels as Thermochromic Materials. *ACS Appl. Energy Mater.* **4**, 9783-9791 (2021).

32. Xu, W. et al. A one-step procedure for elevating the quality of konjac flour: Azeotropy-assisted acidic ethanol. *Food Hydrocolloids* **35**, 653-660 (2014).

33. Weißenborn, E. & Braunschweig, B. Hydroxypropyl cellulose as a green polymer for thermo-responsive aqueous foams. *Soft Matter* **15**, 2876-2883 (2019).

34. Entezari, A., Ejeian, M. & Wang, R.Z. Super Atmospheric Water Harvesting Hydrogel with Alginate Chains Modified with Binary Salts. *ACS Mater. Lett.* **2**, 471-477 (2020).

35. Kim, H. et al. Adsorption-based atmospheric water harvesting device for arid climates. *Nat. Commun.* **9**, 1191 (2018).

36. Kim, H. et al. Water harvesting from air with metal-organic frameworks powered by natural sunlight. *Science* **356**, 430-434 (2017).

37. Zhao, F. et al. Super Moisture-Absorbent Gels for All-Weather Atmospheric Water Harvesting. *Adv. Mater.* **31**, e1806446 (2019).

38. Si, Y. et al. Ultralight Biomass-Derived Carbonaceous Nanofibrous Aerogels with Superelasticity and High Pressure-Sensitivity. *Adv. Mater.* **28**, 9512-9518 (2016).

39. Guo, Y. et al. Biomass-Derived Hybrid Hydrogel Evaporators for Cost-Effective Solar Water Purification. *Adv. Mater.* **32**, e1907061 (2020).

40. Bagheri, F. Performance investigation of atmospheric water harvesting systems. *Water Resour. Ind.* **20**, 23-28 (2018).
